# Electrophoresis Assembly of Novel Superhydrophobic Molybdenum Trioxide (MoO_3_) Films with Great Stability

**DOI:** 10.3390/ma12030336

**Published:** 2019-01-22

**Authors:** Xiaogang Guo, Taotao Liang

**Affiliations:** 1 Chongqing Key Laboratory of Inorganic Special Functional Materials, College of Chemistry and Chemical Engineering, Yangtze Normal University, Chongqing 408100, China; 2 College of Chemistry and Environmental Engineering, Institute of Functional Materials, Material Corrosion and Protection Key Laboratory of Sichuan Province, Sichuan University of Science and Engineering, Zigong 643000, China; 3 Faculty of Materials and Energy, Southwest University, Chongqing 400715, China; liangtaotao@email.swu.edu.cn

**Keywords:** Schistose molybdenum trioxide, Hydrothermal synthesis, Electrophoretic deposition, high-efficient, superhydrophobic stability

## Abstract

This work presents a hydrothermal synthesis approach to produce novel schistose molybdenum trioxide (MoO_3_) powders with wide application, and introduces a facile electrophoresis assembly technique to construct the superhydrophobic MoO_3_ films (SMFs) with contact angle up to 169 ± 1° at normal pressure and temperature. The microstructures and chemical compositions of product were analyzed by field emission scanning electron microcopy (FESEM), energy dispersive X-ray spectroscopy (EDX), X-ray diffraction (XRD). The wettability and stability studies indicate that the SMFs all show great resistance in various environments with adjusting factors, including droplets with different surface tension, pH, relative humidity, etc., and the stability can be maintained at least for five months. Notably, this paper will provides a valuable reference for designing novel oxide powders and their high-efficient hydrophobic film formation with self-cleaning or water proof properties.

## 1. Introduction

Transition metal oxide materials have received growing interest in fields of sensors, photo-catalysts, energy storage, and conversion devices, amongst others because of their special quantum confinement effect [1,2,3]. Among these materials, molybdenum trioxide (MoO_3_) and MoO_3_-based micro/nano-materials with typical layered crystalline structures and the wide range of van der Waals gap of 2.8–3.6 eV [4], show broad prospect owing to their eco-friendliness, natural abundance, highly electronic conductivity and chemical stability [5,6], and they also possess several outstanding advantages for designing effective flame retardants, electrochromic electrode materials, photochromic, and other devices, via designing their various microstructures, including micro/nano-tubes, micro/nanorods, and micro/nanoplatelets [7,8,9,10,11,12].

Recently, different synthesis techniques have been reported to fabricate MoO_3_ with multiple structures [13,14]. Specifically, the mono- and few-layer MoO_3_ sheets were obtained via a rapid flame synthesis method on the typical 2D layered materials reported by Cai et al. [15]. A controlled solid-state chemical synthesis was used to prepare the bar-like MoO_3_ arrays [16]. Wang et al. reported the method of sublimation to fabricate the ultrafine MoO_3_ powders from the industrial grade MoO_3_ in the temperature range of 1123–1373 K [17]. Chen et al. demonstrated the realization of MoO_3_ nanosheets by using in situ growth on Mo wires in air [18]. Nevertheless, the majority of reported methods are usually intricate with major limitations. For example, thermal evaporation technique typically needs harsh vacuum conditions, and flame synthesis method is usually high-cost and complicated in construction operation process. Recently, hydrothermal synthesis method has been successfully used to grow oxides (R_x_O_y_, R = Zn, Ti, V, Mo etc.) [19,20,21,22,23,24,25], which is regarded as a high-efficient technique with the advantages of low-cost, straightforward operation, and high controllability of the microstructures of target powders or films.

**Figure 1:**
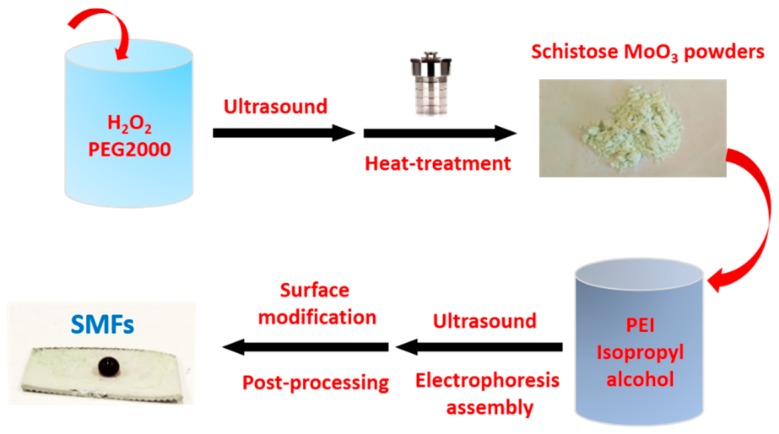
Schematic diagram of facile preparation of novel schistose MoO_3_ powders and SMFs.

Herein, we report a facile route for the synthesis of schistose MoO_3_ powders by using hydrothermal reaction process. Moreover, it is rather interesting to realize film-forming based MoO_3_ powders. Up to now, film-forming techniques including magnetron sputtering [26], atomic layer deposition [27], and spray pyrolysis technique [28] have been reported to obtain MoO_3_ films. However, few reports on electrophoresis assembly of MoO_3_ films and analysis of their anti-wetting properties. In our previous research, we have demonstrated that the electrophoresis assembly is a high-efficient and promising coating-forming method with a rapid deposition rate at NPT (normal pressure and temperature) [29,30,31]. Hence, this paper represents the first attempt for designing superhydrophobic MoO_3_ films (SMFs) with wide potential applications via electrophoresis assembly, which also provides an appropriate, or even optimal assembly process and design concept reference for other oxide systems. The corresponding mechanism diagram is shown in Figure 1. Moreover, the microstructures, superhydrophobicity and anti-wetting properties of SMFs are systematically analyzed in the following sections.

## 2. Materials and Methods

### 2.1. Reagents and Materials

1H, 1H, 2H, 2H-perfluorodecyltriethoxysilane, polyethyleneimine (PEI), and Mo (99.5%) were purchased from Aladdin Industrial Corporation (Shanghai, China). Isopropyl alcohol was purchased from Kelong Industrial Inc., Chengdu, China. The other reagents purchased from Sinopharm Chemical Reagent Co., Ltd. Shanghai, China were used as received without further purification.

### 2.2. Synthesis of Schistose MoO_3_ Powders

A one-step synthesis process was developed to fabricate novel schistose MoO_3_ powders. To be specific, a certain amount (0.1 M) of molybdenum (Mo) powder was added into 80 mL deionized water with trace amount of additive PEG2000 in a beaker, and then a small amount of H_2_O_2_ with a concentration of 30% was added into the above mixture drop by drop until the occurrence of the orange molybdenum peroxide sol. Then, the obtained sol was sonicated for 0.25 h by an ultrasonic generator with 150 W, and transferred into a 100 mL Teflon-lined stainless steel autoclave, which was screwed and heated at 483 ± 2 K for 4 h. At last, the targeted MoO_3_ powders were obtained after undertaking several processes of natural cooling in air, centrifugal cleaning with ethanol and deionized water for five respective times, followed by vacuum drying at 373 K for 1 h.

### 2.3. Electrophoresis Assembly of SMFs

To fabricate the superhydrophobic MoO_3_ films (SMFs), the electrophoresis assembly technique was used in this section. The obtained MoO_3_ powders were added into a mixture of 100 mL isopropyl alcohol and 0.01 mL PEI as the additive, and then sonicated for 25 min in NPT. The commercial Ti sheet (99%) was utilized as electrode material after it was polished to light, washed by ethanol and deionized water and dried under vacuum, successively, and used for the following fabrication step of SMFs. Then, an applied field strength (E) was set as 15 V/mm during electrophoresis assembly of samples, and the distance between two electrode materials was controlled at 10 mm. After assembly process, electrophoretic MoO_3_ films were moved into an oven for drying treatment. Then, SMFs were obtained after surface modification process of MoO_3_ films by soaking in a mixture of ethanol/FAS-17 with volume ratio of 100:1 at 323 K for 2 h, and heat treatment in a vacuum drying oven for 0.4 h to remove surface impurities, followed by natural cooling in the vacuum oven. 

### 2.4. Characterization

The microstructures of SMFs were investigated by using field emission scanning electron microscope (FESEM, JSM-7800F, Tokyo, Japan). The surface composition analysis of samples was carried out using an X-ray diffractometer (XRD-6000, Shimadzu, ZD-3AX, Inc., Tokyo, Japan) and energy-dispersive X-ray spectroscope (EDS, IUCA Energy, Tokyo, Japan). The anti-wetting properties were determined using an optical contact angle meter (HARKE-SPCA, Beijing, China) at NPT (normal pressure and temperature) and a digital camera (D7000, Nikon, Tokyo, Japan). The salt spray test chamber (YWX/Q, YSL, Inc., Beijing, China) was used to explore stability of SMFs.

## 3. Result and Discussion

### 3.1. Characterization of the Schistose MoO_3_ Powders and Target Film-SMFs

**Figure 2:**
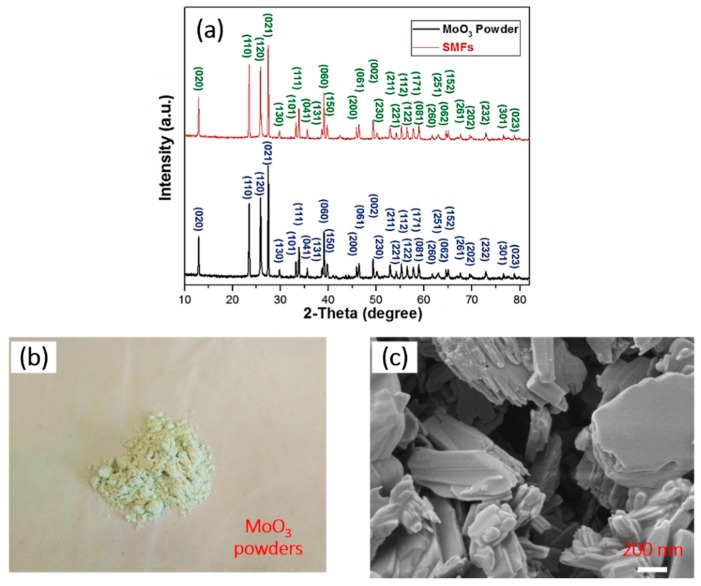
(**a**) The typical XRD spectra of the MoO_3_ powders and SMFs, the optical photograph (**b**) and FESEM image (**c**) of the schistose MoO_3_ powders.

Figure 2a shows the XRD patterns of the schistose MoO_3_ powders and SMFs prepared by hydrothermal synthesis method and electrophoresis assembly at NPT, respectively. Clearly, all mainly diffraction peaks with intensities are indexed, indicating the high purities of samples. As for schistose MoO_3_ powders, the sharp diffraction peaks located at different 2θ (seen in Table S1 in Supporting Information) prove a close match with the relevant standards of MoO_3_ [JCPDS Card No. 35-0609, Space group of Pbnm (62)], which turns out to be the successful fabrication of MoO_3_ white powders as displayed in Figure 2b. Compared with the XRD results of the MoO_3_ powders, the diffraction peaks on SMFs are almost located at the same degree in Figure 2a, showing the successful electrophoresis assembly of MoO_3_ powders forming target film-SMFs. Moreover, Figure 2c displays the typical FESEM image of schistose MoO_3_ powders. It is obviously observed that there are promising schistose structures on MoO_3_ powders, which contributes to constructing rough structures that are the essential principle for designing hydrophobic materials [31,32,33], to realize the superhydrophobicity of SMFs.

**Figure 3:**
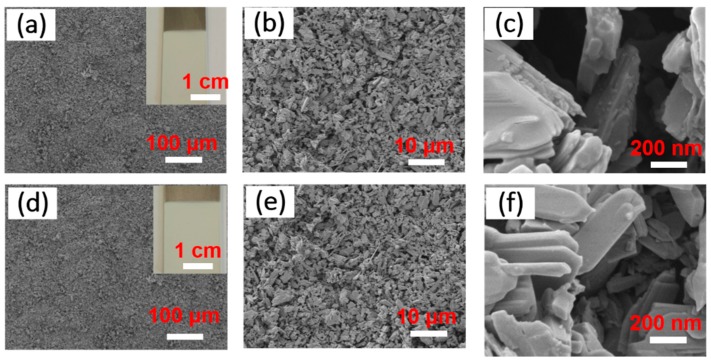
The typical FESEM images of product samples before (**a**), high resolution: (**b**) and (**c**) and after (**d**), high resolution: (**e**) and (**f**) surface modification. The image embedded in (**a**) and (**d**) shows their respective optical photograph.

The microstructures of samples are clearly seen in Figure 3. The schistose MoO_3_ powders have been assembled into their corresponding uniform film by electrophoresis assembly technique, as shown in Figure 3a, indicating the isopropyl alcohol and PEI turning out to be a suitable dispersing agent for this system. The embedded photo in Figure 3a shows the macroscopic optical image of samples without surface modification. The higher resolution FESEM image of samples (seen in Figure 3b) displays the special abundant rough microstructures, providing the structural foundation for constructing superhydrophobic coatings. In addition, the obtained MoO_3_ powders turn out to be schistose-like, clearly seen in Figure 3c. Figure 3d–f display the microstructures of SMFs after surface modification by ethanol/FAS-17. Compared with samples before undergoing surface treatment, the SMFs remain evenly distributed and possess the similar rough internal textures with a great number of micro/nano-wall layers consisting of MoO_3_ particles and a mass of irregular intervals among them (Figure 3e,b). Moreover, that special structures largely contribute to capturing or adsorption of gas in surperhydrophobic materials (e.g., SMFs).

**Figure 4:**
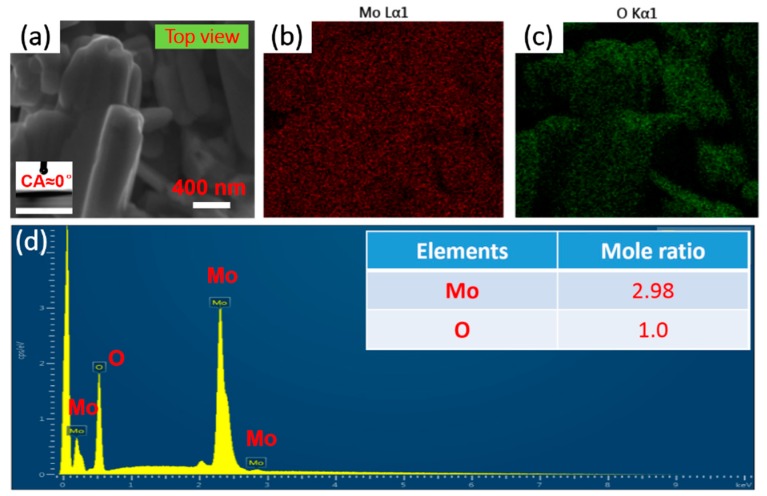
(**a**) The top-view images of samples before surf ace modification. Following are the elemental distributions of (**b**) Mo and (**c**) O, and the EDX spectrums (**d**) of the samples.

**Figure 5:**
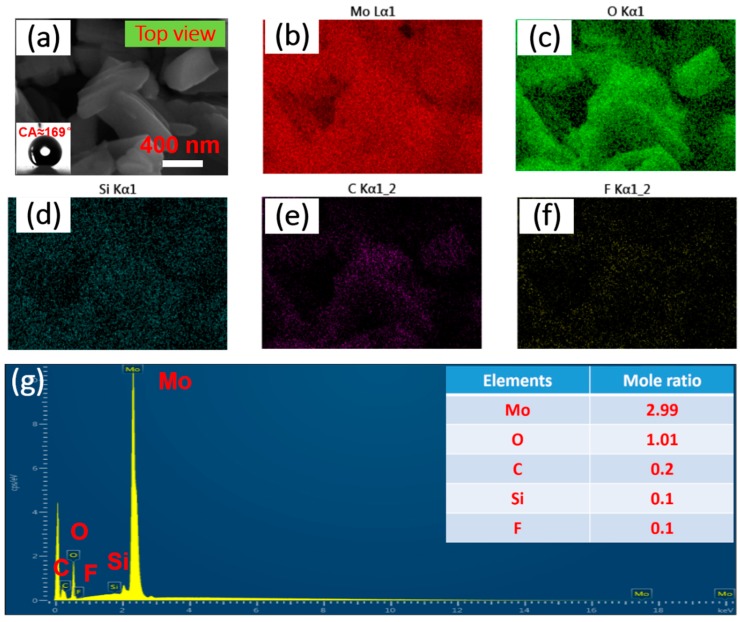
(**a**) The top-view images of SMFs. Following are the elemental distributions of (**b**) Mo, (**c**) O, (**d**) Si, (**e**) C and (**f**) F and the EDX spectrums (**g**) of the samples. The entire region in the top-view image is the elemental mapping region.

In addition, the uniformity is also clearly displayed in the element distribution diagrams by energy dispersive X-ray spectroscopy (EDX), the results of which are based on samples before and after surface modification shown in Figure 4 and Figure 5. As for the schistose MoO_3_ films fabricated by electrophoresis assembly method, all the expected elemental signals of Mo and O are shown in Figure 4 b and c from the top-view region in Figure 4a. The entire region in the top-view image is the elemental mapping region. Moreover, the two main peaks of Mo and O in the EDX spectrums (seen in Figure 4d) indicate the element of Mo and O as the composition of the film and the mole ratio of Mo to O is nearly 3:1, demonstrating the successful electrophoresis assembly application to schistose MoO_3_ films. However, as for target superhydrophobic film-SMFs after surface modification, there are five elemental signals, including Mo (Figure 5b), O (Figure 5c), and additional three elements of Si (Figure 5d), F (Figure 5e) and C (Figure 5f), which are also demonstrated in the EDX spectrum results in Figure 5g. The added elements probably come from the modification treatment, that is, the FAS-17 graft process. It is worth mentioning that after surface modification treatment, the samples turns to superhydrophobic from superhydrophilic, due to the relatively uniform rough structures designed by electrophoresis assembly and aid of a modifier with low surface energy, such as FAS-17 [30,32]. As shown in Figure 5g, the mole ratio of Mo and O is 2.99:1.01 (ca. 3:1), which is consistent with the molecular structural formula of MoO_3_ and the EDX results in Figure 4d, showing there is almost no effect of modification process on the composition of materials, and the peaks of the other elements of Si, F and C are weak, and they are low in mole ratios. All results indicate the successful fabrication of SMFs with promising structures.

### 3.2. Wettability Study

**Figure 6:**
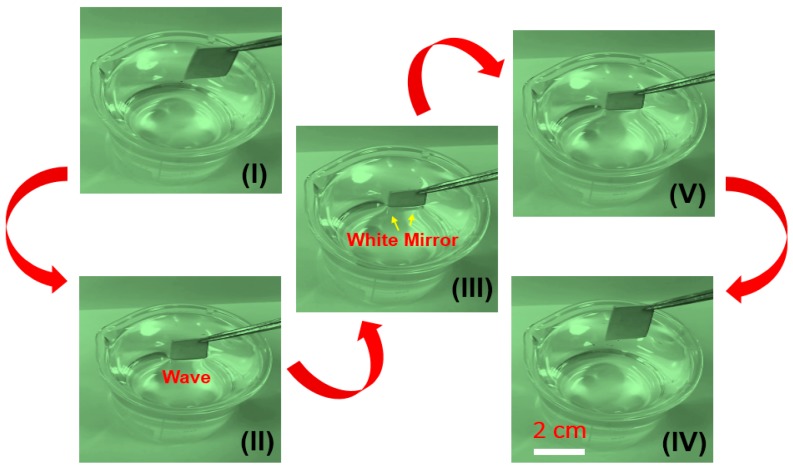
Dynamic immersion test process of SMFs into water and simultaneous high-speed images captured from the side.

**Figure 7:**
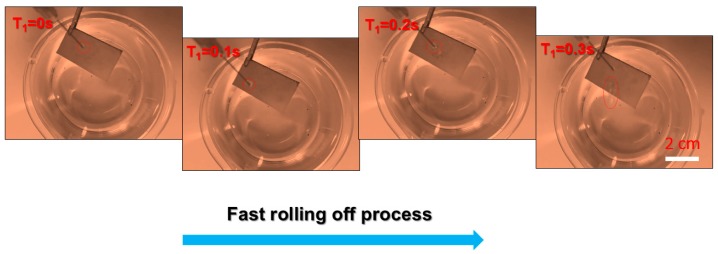
Dynamic process of droplet (d ≈ 2.6 mm) impacting the sample surface and simultaneous high-speed images captured from the side.

The wettability of SMFs is systematically studied for analyzing their anti-wetting performance. As shown in Figure 4a, the contact angle of samples before modification treatment is close to 0° by using a water droplet (V = 10 μL), indicating the superhydrophilic material. However, after modification process, it is quite difficult to place the droplet on the modified samples due to droplets rolling down quickly. The SMFs show great anti-wetting properties with an apparent contact angle of 169 ± 1° and a rather small roll angle of <1 ± 1°, which means the SMFs belong to superhydrophobic materials [33,34,35]. The droplet approximates a sphere (Figure 5a) in the typical Cassie state [36], indicating the outstanding anti-wetting properties of product. Furthermore, Figure 6 shows the dynamic soaking process of SMFs into water, including five steps of falling (I), contacting (II), soaking (III), rising up (IV) and departing (V). Clearly seen step II in Figure 6, when the SMFs contacts with the water, distorted water ripples appear, due to the water-proof characteristic of SMFs, pushing the water away in an interesting way. Notably, when samples is immersed into the water, it appears as a silver-white mirror, which is consistent with reported by Larmour group [37]. The high absolute reflectivity and mirror-like appearance is because of an air layer between the sample surface and water. The SMFs are always dry after immersion experiments, which is also demonstrated in Video SI in Supporting Information. Moreover, the water droplet can roll off quickly with a contact time of less than 0.3 s when placed on SMFs’ surface with a negligible angle or an almost flat state, clearly seen in Figure 7. The corresponding whole video is displayed in Video SII in Supporting Information.

**Figure 8:**
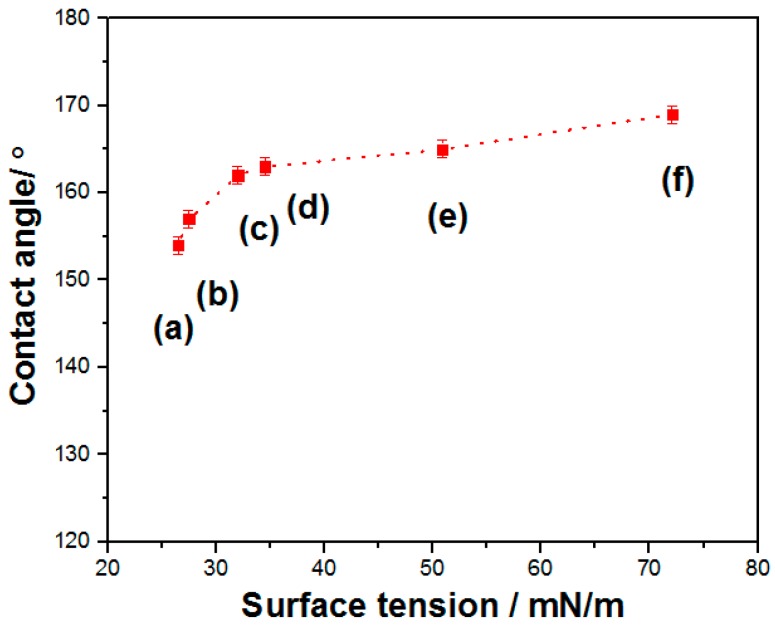
The relationship of the apparent contact angle contact angle of products to droplets with different surface tensions.

As we all known, all materials will contact with different kinds of external environments in practical fields. Furthermore, in order to verify the practicality of SMFs, droplets ((a) tetradecane, (b) hexadecane, (c) olive oil, (d) peanut oil, (e) diiodomethane, and (f) water) with different surface tensions are used to investigate wettability of products. Figure 8 shows the contact angle of SMFs as a function of droplets with various surface tensions. Clearly, contact angle gradually decreases as the surface tension becomes larger. When the drops with organic liquid are placed on the surface of SMFs, the apparent contact angle changes from 166° for diiodomethane to 154° even for tetradecane with surface tension of ca. 26 mN/m, indicating the outstanding anti-wetting properties of product.

### 3.3. Stability Analysis

**Figure 9:**
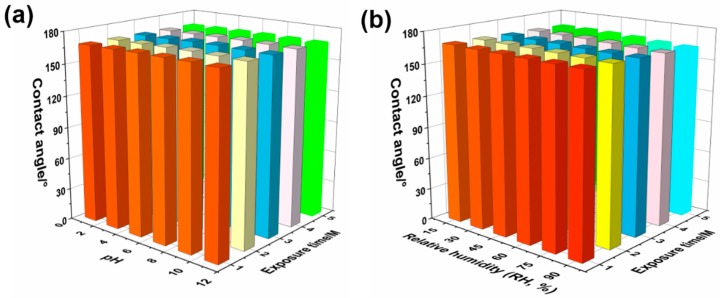
The contact angle as functions of (**a**) pH and exposure time and (**b**) relative humidity and exposure time, respectively.

The effect of different environments on the superhydrophbicity of SMFs are investigated in detail by adjusting pH, exposure time and relative humidity (RH). Figure 9a shows the contact angle as functions of pH and exposure time. It is obviously seen that the contact angle increases slightly as pH rises up to 7, and decreases when pH continues to grow when exposure time is one month. However, the apparent contact angle remains more than 165° regardless of how the pH changes. When the exposure time increases from one to five months, the apparent contact angle changes in a rather narrow range. Similarly, the change laws of contact angle occur on environments with different pH, and samples still show great superhydrophobicity with an apparent contact angle of >159 ± 1° even in the strong alkali (pH = 11) or strong acid (pH = 1). Moreover, the relationship of RH and exposure time to contact angle is displayed in Figure 9b. Clearly, there are negligible fluctuations on the contact angle of SMFs with RH changing from 15% to 90% when the exposure time is controlled at one month. Additionally, as exposure time increases to five months, the apparent contact angle of samples nearly remains stable as 167 ± 1°, as RH is the maximum value of 90%. Thus, all results indicate that the obtained novel SMFs by electrophoresis assembly possess the great superhydrophobicity and promising potential applications in many fields. 

## 4. Conclusions

In brief, novel schistose MoO_3_ powders with promising application have been synthesized by a facile hydrothermal reaction method, and the corresponding film-SMFs with uniform distribution are achieved by a straightforward coating method of electrophoresis assembly technique. The wettability and stability studies show great superhydrophobicity or anti-wetting properties even using low-surface-tension drops of tetradecane, and the robust resistance of SMFs to various environments for long times, up to five months, shedding new light on the research of various novel oxides or their superhydrophobic films in various fields.

## Reference

## Supplementary Materials

Supplementary materials are available online at http://www.mdpi.com/1996-1944/12/3/336/s1. Table S1: the detailed data of XRD main peaks for schistose MoO_3_ powders and SMFs. Video SI: The immersion test process of SMFs into aqueous solution in 100 mL beaker, the surface of samples can not be wetted, suggesting the great superhydrophobicity of SMFs. Video SII: The droplet impacting experiment of SMFs with a negligible angle or an almost flat state fixed by using a tweezer.
