# Supplementary material for: Electrophoresis Assembly of Novel Superhydrophobic Molybdenum Trioxide (MoO3) Films with Great Stability
Source: Materials (Basel). 2019 Jan 22;12(3):336. doi: 10.3390/ma12030336 (PMC6384839; doi:10.3390/ma12030336)
Supplement: Supplementary file 1 [file materials-12-00336-s001.zip › Supplementary Materials.docx]

Article

Electrophoresis Assembly of Novel Superhydrophobic Molybdenum Trioxide (MoO_3_) Films with Great Stability

Xiaogang Guo ^1,2,^* and Taotao Liang ^3^

^1^ Chongqing Key Laboratory of Inorganic Special Functional Materials, College of Chemistry and Chemical Engineering, Yangtze Normal University, Chongqing 408100, China

^2^ College of Chemistry and Environmental Engineering, Institute of Functional Materials, Material Corrosion and Protection Key Laboratory of Sichuan Province, Sichuan University of Science and Engineering, Zigong 643000, China

^3^ Faculty of Materials and Energy, Southwest University, Chongqing 400715, China; liangtaotao@email.swu.edu.cn

***** Correspondence: guoxiaogang0528@126.com

Received: 5 December 2018; Accepted: 2 January 2019; Published: 22 January 2019

**Table S1.** The detailed data of XRD main peaks for schistose MoO_3_ powders and SMFs.

| **(2θ)**  **2-Theta** | **The diffraction plane** | **h** | **k** | **l** |
| --- | --- | --- | --- | --- |
| 12.78 | 020 | 0 | 2 | 0 |
| 23.339 | 110 | 1 | 1 | 0 |
| 25.879 | 120 | 1 | 2 | 0 |
| 27.339 | 021 | 0 | 2 | 1 |
| 29.679 | 130 | 1 | 3 | 0 |
| 33.119 | 101 | 1 | 0 | 1 |
| 33.759 | 111 | 1 | 1 | 1 |
| 35.499 | 041 | 0 | 4 | 1 |
| 38.559 | 131 | 1 | 3 | 1 |
| 38.979 | 060 | 0 | 6 | 0 |
| 39.659 | 150 | 1 | 5 | 0 |
| 45.759 | 200 | 2 | 0 | 0 |
| 46.319 | 061 | 0 | 6 | 1 |
| 49.259 | 002 | 0 | 0 | 2 |
| 50.059 | 230 | 2 | 3 | 0 |
| 52.799 | 211 | 2 | 1 | 1 |
| 54.119 | 221 | 2 | 2 | 1 |
| 55.199 | 112 | 1 | 1 | 2 |
| 57.678 | 171 | 1 | 7 | 1 |
| 58.839 | 081 | 0 | 8 | 1 |
| 61.618 | 260 | 2 | 6 | 0 |
| 62.838 | 251 | 2 | 5 | 1 |
| 64.518 | 062 | 0 | 6 | 2 |
| 64.998 | 152 | 1 | 5 | 2 |
| 67.138 | 261 | 2 | 6 | 1 |
| 69.478 | 202 | 2 | 0 | 2 |
| 72.838 | 232 | 2 | 3 | 2 |
| 76.518 | 301 | 3 | 0 | 1 |
| 78.838 | 023 | 0 | 2 | 3 |

**Video S1: The immersion test process of** **SMFs into aqueous solution in 100 mL beaker, the surface of samples can’t be wetted suggesting the great superhydrophobicity of SMFs.**

**Video S2: The droplet impacting experiment of SMFs with a negligible angle or an almost flat state fixed by using a tweezer.**

© 2019 by the authors. Licensee MDPI, Basel, Switzerland. This article is an open access article distributed under the terms and conditions of the Creative Commons Attribution (CC BY) license (http://creativecommons.org/licenses/by/4.0/).
